# Renal disease pathophysiology and treatment: contributions from the rat

**DOI:** 10.1242/dmm.027276

**Published:** 2016-12-01

**Authors:** Linda J. Mullins, Bryan R. Conway, Robert I. Menzies, Laura Denby, John J. Mullins

**Affiliations:** University of Edinburgh/British Heart Foundation Centre for Cardiovascular Science, Queen's Medical Research Institute, 47 Little France Crescent, Edinburgh EH16 4TJ, UK

**Keywords:** Rat, Chronic kidney disease, Diabetic nephropathy, Genetically modified rats, End-organ damage, Renal transplantation

## Abstract

The rat has classically been the species of choice for pharmacological studies and disease modeling, providing a source of high-quality physiological data on cardiovascular and renal pathophysiology over many decades. Recent developments in genome engineering now allow us to capitalize on the wealth of knowledge acquired over the last century. Here, we review rat models of hypertension, diabetic nephropathy, and acute and chronic kidney disease. These models have made important contributions to our understanding of renal diseases and have revealed key genes, such as *Ace* and *P2rx7*, involved in renal pathogenic processes. By targeting these genes of interest, researchers are gaining a better understanding of the etiology of renal pathologies, with the promised potential of slowing disease progression or even reversing the damage caused. Some, but not all, of these target genes have proved to be of clinical relevance. However, it is now possible to generate more sophisticated and appropriate disease models in the rat, which can recapitulate key aspects of human renal pathology. These advances will ultimately be used to identify new treatments and therapeutic targets of much greater clinical relevance.

## Abstract (precis)

**Summary:** This Review highlights the key role that the rat continues to play in improving our understanding of the etiologies of renal pathologies, and how these insights have opened up new therapeutic avenues.

## Introduction

The prevalence of chronic kidney disease (CKD) is estimated to be 8-16% worldwide (Jha et al., 2013; Stevens et al., 2007). With an aging population, and rising levels of hypertension, diabetes and obesity, renal diseases pose an increasing burden on public healthcare. Two million people worldwide are currently on renal replacement therapy (RRT), dialysis or have a renal transplant. However, this figure makes up only ∼10% of all individuals who actually need RRT, with a greater number dying due to the inadequate availability of therapies (https://www.kidney.org/kidneydisease/global-facts-about-kidney-disease#_ENREF_3) and skewed treatment towards affluent countries with access to healthcare (Jha et al., 2013). Furthermore, kidney disease represents an independent risk factor for cardiovascular mortality (Tonelli et al., 2006). Individuals often present with complex renal pathologies resulting from numerous insults, both genetic and environmental. The interactions of combined metabolic and cardiovascular factors make it difficult to identify individuals who will benefit most from available treatments to slow or prevent disease progression (Jha et al., 2013). It is therefore imperative that we develop new strategies to identify those at high risk of progressive kidney disease and to discover new therapies to slow the rate of disease progression in these individuals. Animal models can provide insight into the pathophysiology of kidney disease and can be used to test novel therapies. However, their utility is limited by how well they recapitulate the key features and mechanisms of progressive human disease. Although it can be argued that rodents are poor replacements for humans in studies of kidney disease (Becker and Hewitson, 2013), much valuable information about the underlying etiology of renal disease has been revealed by studying rat models.

**Fig. 1.**
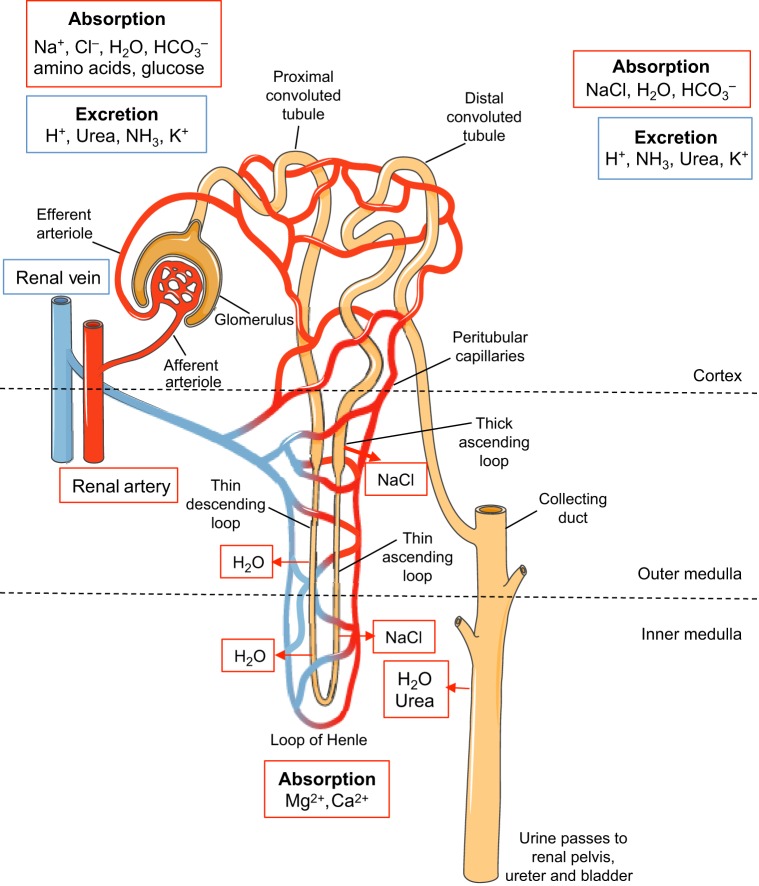
**Schematic of a nephron.** This schematic shows a nephron, the functional unit of the kidney. Blood is delivered to the glomerulus, where plasma is filtered into the lumen of the tubule. Various ions are excreted and absorbed, and water is retrieved, as plasma passes through the different segments of the tubule, which are intimately linked to peritubular capillaries. Concentrated urine is formed by this filtration process, which then passes through the collecting duct to the renal pelvis. The different components of a nephron occupy distinct regions of the kidney: the cortex and outer and inner medulla, as shown.

The functional unit of the kidney is the nephron (see Glossary, Box 1), which is closely integrated with the renal blood supply (Fig. 1). The human kidney filters 180 liters of plasma through its glomeruli, and produces 1 to 2 liters of urine daily. Approximately 99% of filtered sodium is retrieved as it passes through various sections of the nephron before reaching the collecting duct.

**Box 1. Glossary**

**Albuminuria:** high levels of albumin (protein) in the urine.

**Arteriolar hyalinosis:** the thickening of the arteriole wall with proteinaceous deposits of pink-staining hyaline material.

**Capillary rarefaction:** a reduction in capillary density.

**Chronic allograft nephropathy (CAN):** a leading cause of kidney transplant failure; it features a gradual decline in kidney function, often with an associated increase in blood pressure.

**Congenic:** a rat strain that carries part of a chromosome from another, different rat strain.

**Consomic:** when two rat strains carry the same transgene inserted at the same place in the genome.

**Cre recombinase/*loxP*:** Cre recombinase enzymatically removes sequences that are flanked (floxed) by inserted *loxP* sequences.

**CRISPR-Cas9:** a genome-engineering technique. CRISPR stands for clustered regularly interspaced short palindromic repeats, which, together with trans-activating guide RNAs, target the sequence-specific double-stranded breakage of DNA by the bacterial protein Cas9 endonuclease.

**Diabetic nephropathy (DN):** a progressive form of kidney disease in diabetics, characterized by albuminuria, a >50% decline in glomerular filtration rate (GFR), increased glomerular basement-membrane thickness, arteriolar hyalinosis, mesangial sclerosis and tubulointerstitial fibrosis.

**Embryonic stem cells (ES cells):** pluripotent stem cells derived from the inner cell mass of a blastocyst, an early-stage preimplantation embryo.

**End-organ damage:** damage occurring in the major organs fed by the circulatory system.

**Extracellular matrix (ECM):** a proteinaceous matrix laid down outside the cell.

**Focal segmental glomerulosclerosis:** the deposition of excess ECM in a subset of glomeruli with only part of each glomerulus affected.

**Glomerular filtration rate (GFR):** the rate at which plasma is filtered through the glomerulus.

**Glomerulosclerosis:** the deposition of excess ECM in the glomerulus.

**Hyperglycemia:** abnormally increased sugar content in the blood.

**Hyperkalemia:** abnormally high potassium concentration in the blood.

**Hypokalemia:** abnormally low potassium concentration in the blood.

**Ischemia-reperfusion injury (IRI):** the tissue damage caused when blood supply returns to the tissue after a period of ischemia or lack of oxygen.

**Malignant hypertension:** a rapid and severe increase in blood pressure, leading to end-organ damage.

**Mesangio-proliferative glomerulonephritis (MPGN):** an autoimmune, inflammatory condition that damages the membrane supporting capillary loops of the glomerulus.

**Mineralocorticoid receptor (MR):** a steroid-responsive nuclear receptor that controls fluid homeostasis in the kidney; it also has pro-inflammatory and pro-proteinuric effects.

**Myofibroblast:** a cell that combines the ultrastructural features of a fibroblast and a smooth-muscle cell.

**Nephron:** the functional unit of the kidney, consisting of the proximal tubule, the loop of Henle, and the distal convoluted tubule, each lined with specialized tubular epithelial cells that express ion channels and transporters.

**Nocturnal dipping:** when systolic blood pressure falls by more than 10% at night compared to daytime levels.

**Pericyte:** contractile cell that wraps around the endothelial cells of capillaries and venules throughout the body.

**Podocyte:** a modified epithelial cell of the glomerulus that has foot-like processes, which contact the basal lamina of glomerular capillaries and allow blood to filter through the slits.

**Pressure-diuresis response:** the increase in urine output for a given imposed increase in blood pressure.

**Renin-angiotensin aldosterone system (RAAS):** a hormone system involved in regulating sodium reabsorption from nephrons and blood pressure.

**Tubulointerstitial fibrosis:** the deposition of collagen in the interstitial region between tubules.

Acute kidney injury (AKI) occurs when there is a rapid decline in glomerular filtration rate (GFR; see Glossary, Box 1), usually accompanied by impaired microcirculation, inflammation and/or tubular injury or necrosis and reduced renal blood flow (Basile et al., 2012). AKI is initiated by various clinical insults, including hypotensive shock, sepsis, surgery or the administration of nephrotoxic agents such as cisplatin (Tanaka et al., 2005) and contrast agents (commonly used for medical imaging) (Mehran and Nikolsky, 2006). Following mild kidney injury, an adaptive repair response might ensue, leading to kidney regeneration. However, with more severe injury, regeneration is incomplete and nephron mass can be replaced by scar tissue, leading to CKD (Bucaloiu et al., 2012; Chawla et al., 2011). There are limited treatment options available for AKI, and its associated mortality remains high (Ferenbach and Bonventre, 2015). AKI can be induced in rats by performing ischemia-reperfusion surgery or by administering toxins such as cisplatin. However, these single insults are unlikely to fully recapitulate the multiple injurious processes that have typically occurred in individuals with AKI.

**Fig. 2.**
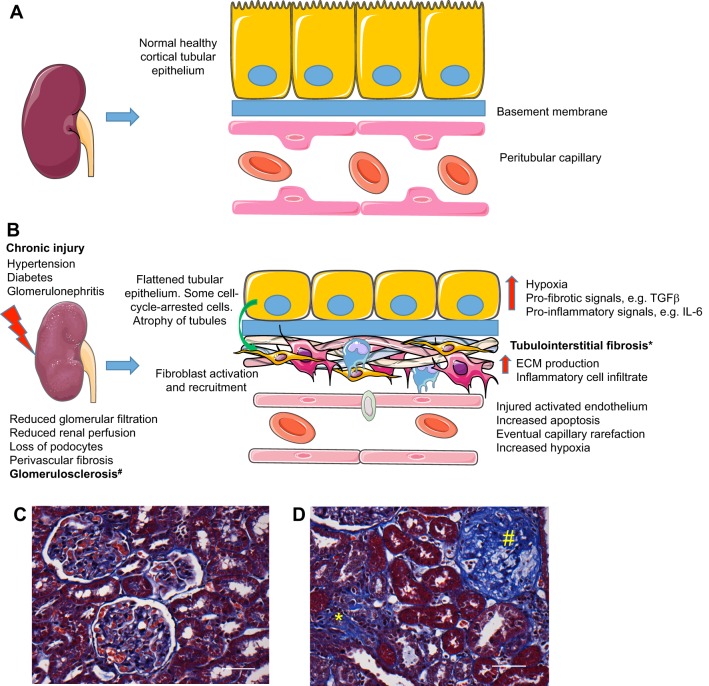
**The pathophysiological processes linked to kidney disease.** (A) A normal, healthy kidney (left), and a magnified view of the structure of a tubule and its associated vasculature (right). (B) A chronically diseased kidney, showing the processes that lead to tubulointerstitial fibrosis. (C,D) Histological sections of an adult rat kidney, stained with Masson's trichrome (20× magnification; scale bars: 50 µm). (C) The glomerular and tubular architecture of a normal adult rat kidney, and (D) glomerulosclerosis (#) and tubulointerstitial fibrosis (*) in a 12-month-old hydroxysteroid dehydrogenase 2 (*Hsd11b2*)-knockout rat exhibiting end-stage renal disease.

CKD is an umbrella term for any renal disease that results in the progressive loss of kidney function over time. The kidney possesses only a limited capacity for regeneration, and repeated or sustained injury to the kidney results in maladaptive responses (Ferenbach and Bonventre, 2015), including the deposition of excess extracellular matrix (ECM; see Glossary, Box 1), particularly collagen, in the glomerulus and tubulointerstitium of the kidney (Fig. 2). The pathological changes associated with CKD include glomerulosclerosis and tubulointerstitial fibrosis (see Glossary, Box 1), which result in the loss of normal renal architecture, microvascular capillary rarefaction (see Glossary, Box 1), hypoxia and tubular atrophy. These changes lead to the loss of renal filtrative capacity and ultimately to end-stage renal disease. Many rodent models mimic features of early CKD; however, only few exhibit features of end-stage renal disease (ESRD).

The substantial wealth of physiological knowledge available for the rat makes it the species of choice for modeling aspects of kidney disease and for exploring therapeutic strategies *in vivo*. For several decades, the mouse has been the pre-eminent mammalian organism for disease modeling because of its genetic tractability. With recent developments in genome engineering, the rat is rapidly catching up. Genetic, congenic, transgenic, knockout, surgical or pharmacological rat models have provided an opportunity to investigate the molecular pathogenesis of renal disease, to examine the disease in the context of live animals, and to assess potential novel therapies. Table 1 lists the rat models (with key genotypic and phenotypic features) discussed in this Review. The interested reader is also directed to the Rat Genome Database (http://rgd.mcw.edu/) for further information about these and additional models (Shimoyama et al., 2016).

**Table 1.**
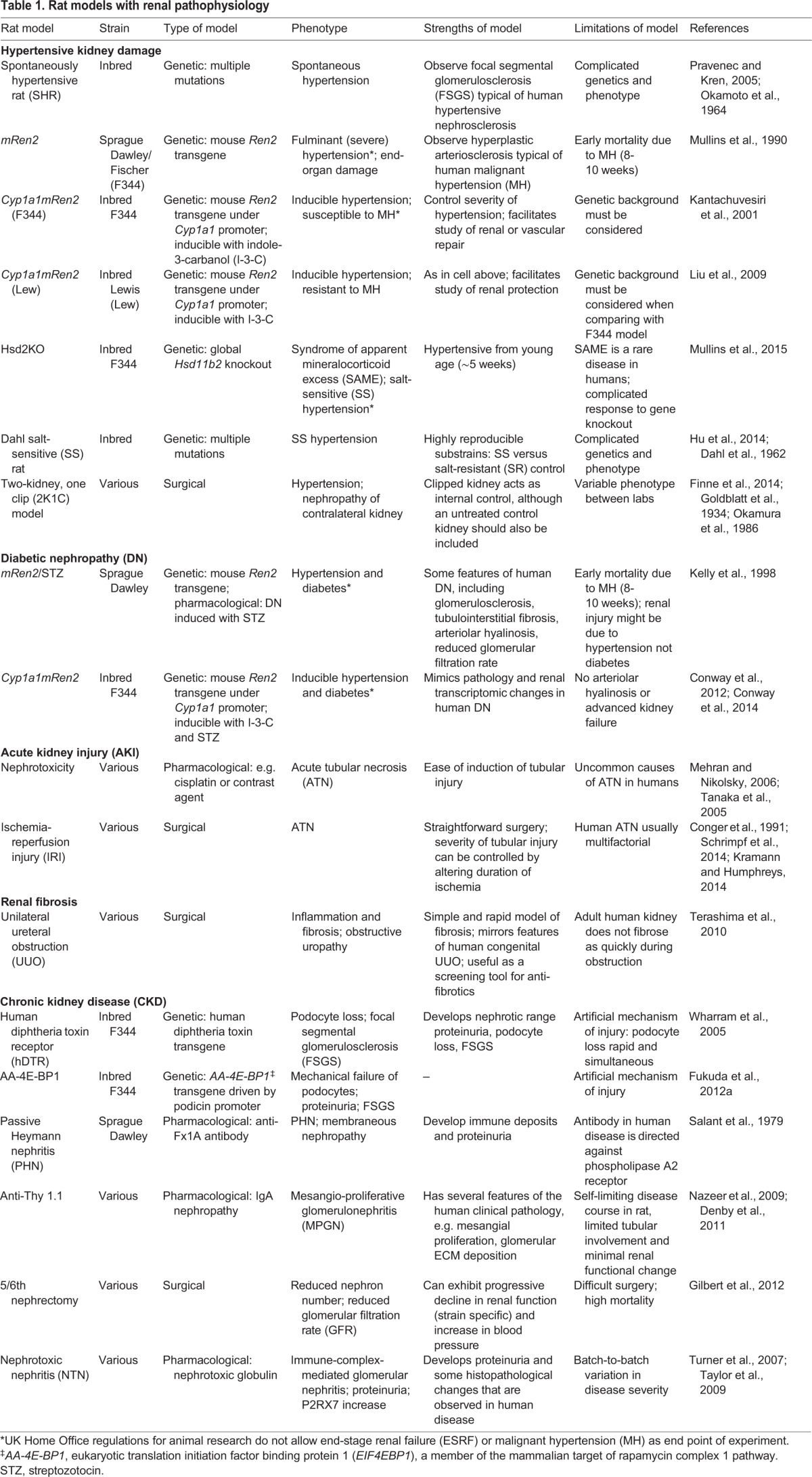
**Rat models with renal pathophysiology**

In this Review, we discuss how rat models have contributed to our understanding of renal pathophysiology and hold promise for developing improved treatments to halt the progression of CKD or to repair kidney damage in humans. We consider aspects of hypertensive renal damage, diabetic nephritis, AKI and CKD. We emphasize the utility and limitations of the rat in recapitulating features of human renal pathologies *in vivo* and how this model organism has shed light on complex underlying mechanisms of disease progression of therapeutic relevance – information that might ultimately lead to the development of new drug treatments and targets (Aitman et al., 2016, 2008).

## Models of hypertensive renal damage

In up to 95% of individuals with hypertension, no specific underlying genetic cause for the condition is identified despite contributory factors such as smoking or obesity. However, in a small proportion of cases, hypertension is secondary to endocrine or renal disease. Sustained exposure to high blood pressure adversely affects cardiac, brain, vascular and renal tissues, making hypertension a major cause of end-organ damage (see Glossary; Box 1). Hence, renal disease might be both a cause and consequence of hypertension, forming a vicious circle whereby hypertension causes kidney damage, which then exacerbates the high blood pressure. Hypertensive nephrosclerosis is characterized by arterial wall thickening, loss of renal autoregulation, glomerulosclerosis, tubular atrophy and interstitial fibrosis (Hill, 2008). Arterial stiffening due to increased pulse pressure affects autoregulation of the preglomerular afferent arterioles, and leads to progressive glomerular hypertrophy and damage with atrophy of the attached tubule. Reduced glomerular filtration causes compensatory hyperfiltration in other glomeruli, leading to glomerulosclerosis (which also results from ischemic damage) and ultimately to tubular damage and fibrotic lesions of the interstitial cells (Hill, 2008).

Classically, genetic animal models of high blood pressure, such as the spontaneously hypertensive rat (SHR) and the related salt-loaded stroke-prone (SHRSP) rat, generated by protracted rounds of breeding and selection for high blood pressure (see also Table 1), have been used to study the effects of chronic hypertension (Okamoto and Aoki, 1963; Okamoto et al., 1964; Pravenec and Křen, 2005). It has been proposed that the pathological progression of hypertensive damage to kidney damage in this rat model mirrors that seen in human hypertension (Hultström, 2012), with renal damage resulting from altered pressure-dependent autoregulation of renal blood flow.

The underlying mutations and their homeostatic sequelae, which contribute to hypertension and to multi-end-organ damage in the SHR, seem to be very complex. Renal microarray has identified >200 genes that differ more than fourfold in their levels of expression between adult SHRs or SHR substrains (Watanabe et al., 2015) and Wistar Kyoto control rats. The availability of the entire SHR genome sequence (Atanur et al., 2010) provides an opportunity to identify potentially causative polymorphisms in these genes. Undoubtedly, strains such as the SHR have helped to confirm the involvement of multiple genes in hypertension and kidney damage. However, identifying which mutations are primary and which are secondary to the disease remains an unresolved question for cardiovascular research.

Transgenesis allows researchers to investigate the biological consequence(s) of a genetic perturbation. However, elucidating the homeostatic effects of altered gene function is not always straightforward, as exemplified by the *mRen2* rat (Mullins et al., 1990), which overexpresses the mouse renin (*Ren2*) gene, causing severe hypertension (see Table 1). Renin is a key component of the renin-angiotensin aldosterone system (RAAS; see Glossary, Box 1), the activation of which increases levels of circulating angiotensin II (AngII), and causes systemic vasoconstriction and sodium resorption in the kidney in order to increase blood pressure. Both kidney and plasma levels of renin are low in the *mRen2* rat (Bachmann et al., 1992) making this a low-renin hypertension model. Hypertension was attenuated with captopril, which inhibits the RAAS component angiotensin-converting enzyme (Ace), indicating AngII dependence (Bader et al., 1992). High levels of mouse-transgene-derived inactive renin, and low levels of active renin, were produced in the adrenal gland, indicating that tissue RAAS is responsible for hypertension in this model (Peters et al., 1993). The crossing of the renin transgene onto a closely related outbred Sprague Dawley strain generated animals that developed malignant hypertension and end-organ damage by 8 weeks of age (see Glossary, Box 1) (Whitworth et al., 1994). In particular, the kidney exhibited glomerulosclerosis and interstitial fibrotic lesions. When the *mRen2* transgene was crossed onto the inbred Fischer (F344) and Lewis rat strains, the resulting consomic strains (see Glossary, Box 1) were susceptible and resistant to malignant hypertension, respectively. Genome-wide screening and quantitative trait analysis identified two modifier loci on chromosomes 10 and 17, which contributed to malignant hypertension susceptibility (Kantachuvesiri et al., 1999). The *mRen2* rat strains have been studied extensively for over 25 years, under both hypertensive and hyperglycemic conditions.

In a more refined model, the *Cyp1a1Ren2* rat (Kantachuvesiri et al., 2001), expression of the *mRen2* gene is under the control of an inducible promoter in the inbred Fischer strain. This allows the researcher to control the degree of AngII-dependent hypertension and consequent end-organ damage, its speed of attainment and, also, to look at repair processes, once the inducer (indole-3-carbinol; I-3-C) is withdrawn (see ‘Models of diabetic nephropathy’ below). The earliest hypertension-induced renal injury identified in the *Cyp1a1Ren2*.Fischer strain is limited to the preglomerular vasculature (Ashek et al., 2012). The later-onset hypertensive kidney damage includes arterial wall thickening, glomerulosclerosis, interstitial fibrosis and tubular injury (Kantachuvesiri et al., 2001) similar to the renal damage caused by hypertension in humans. Increases in urinary albumin and angiotensinogen were observed with malignant hypertension (Milani et al., 2010), although the latter did not reflect changes in angiotensinogen gene expression in the kidney cortex (Prieto et al., 2011). Proteinuria was alleviated in this model by antagonism of the mineralocorticoid receptor (MR; see Glossary, Box 1) with spironolactone (Ortiz et al., 2007). After the transient induction of hypertension, *Cyp1a1Ren2* rats developed salt-sensitive hypertension, which could be attenuated by the superoxide dismutase mimetic tempol, implicating the superoxide anion in the development of salt-sensitive hypertension (Howard et al., 2005).

The *Cyp1a1Ren2* transgene is carried on the Y chromosome and, by crossing the inducible Fischer male to a Lewis female, followed by selective backcrossing of the F1 progeny to Lewis or Fischer animals, congenic lines (see Glossary, Box 1) were derived. These lines retain the transgene and either susceptibility or resistance to end-organ damage, on an otherwise resistant or susceptible background (Kantachuvesiri et al., 1999). Whole-renal, microarray-based, gene-expression profiling studies of the parental and congenic strains revealed genes in the congenic region that were differentially expressed between the parental and congenic strains (Liu et al., 2009). This strategy identified angiotensin-converting enzyme Ace as a principal modifier of hypertension-induced microvascular renal injury in the *Cyp1a1Ren2* rat model (Liu et al., 2009). The C-domain of Ace is thought to mediate blood pressure control through its action on angiotensin I. However, it is now recognized that Ace has other effects, such as cleavage of the naturally occurring tetra-peptide acetyl-N-Ser-Asp-Lys-Pro (AcSDKP) by the N-terminal domain of Ace (Bernstein et al., 2011). AcSDKP has been shown to reverse inflammation, cell proliferation and fibrosis in rat models of hypertension (Liu et al., 2009; Zuo et al., 2013). As predicted, AcSDKP was present at significantly lower levels in the kidneys of the injury-susceptible Fischer rat than in the kidneys of the more protected Lewis rat (Liu et al., 2009).

Microarray-based gene-expression profiling of the congenic Fischer and Lewis kidneys was further used to identify previously unknown candidate genes that might associate with a susceptibility to kidney injury (Menzies et al., 2013). A bioinformatic enrichment analysis identified multiple candidate genes in addition to *Ace*. The second- and third-ranked susceptibility genes were the purine receptors *P2X7* and *P2X4* (Menzies et al., 2013). There are seven P2X receptors in the rat, as in humans. These adenosine-5′-triphosphate-activated cation channels are part of the larger mammalian purine receptor family, which includes G-protein coupled P2Y receptors and adenosine P1 receptors (Ralevic and Burnstock, 1998). Both P2X and P2Y purine receptors have been implicated in preclinical rodent models of hypertension (Menzies et al., 2015b) and kidney disease (Menzies et al., 2016; Ralevic and Burnstock, 1998). In humans, genetic variation that causes the functional impairment of P2X7 is associated with a reduced risk of stroke (Gidlöf et al., 2012). Conversely, P2X4 loss of function is associated with increased pulse pressure (Stokes et al., 2011). The renal pressure-diuresis response (see Glossary, Box 1) of Fischer, but not of Lewis, rats was improved with combined P2X7 and P2X4 receptor antagonism using the dye, Brilliant Blue G (BBG) (Menzies et al., 2013). Renal vascular resistance was unaffected by BBG in Lewis rats, but both blood pressure and vascular resistance decreased in Fischer rats, suggesting that P2X7 might support tonic vasoconstriction in the susceptible strain. Specific P2X7 receptor antagonism using the compound AZ11657312 caused rapid vasodilation. Acute antagonism of the receptor P2X7 in Fischer rats, chronically infused with AngII, significantly improved renal perfusion and tissue oxygenation (Menzies et al., 2015a). Recently, P2X7 receptor antagonism has also been shown to attenuate renal injury in Dahl salt-sensitive rats (Ji et al., 2012).

P2X7 has been implicated in a wide range of neurological, inflammatory and musculoskeletal disorders, in addition to its role in hypertension and renal disease. Clinical trials of P2X7 antagonists in the treatment of inflammatory diseases have shown limited therapeutic benefit to date (Bartlett et al., 2014). Given the large number of splice variants (Cheewatrakoolpong et al., 2005) and disease-related single-nucleotide polymorphisms (SNPs) (Jiang et al., 2013) in the human *P2RX7* gene, a productive future research strategy could be the selective humanization of rats to develop tissue-specific or disease-relevant therapeutic strategies.

In the two-kidney, one clip (2K1C) hypertensive system (Goldblatt et al., 1934), which has been implemented in rats, a clip on the left renal artery activates the RAAS system. Although both kidneys are exposed to an equivalent increase in AngII, only the non-clipped rat kidney shows hypertensive damage (Cervenka et al., 1999). Recently, the non-clipped kidney was found to have increased mRNA, protein and urinary levels of angiotensinogen, suggesting that kidney damage occurs through increased AngII, and that angiotensinogen could be used as an early biomarker of kidney damage (Shao et al., 2016). Exposure of the non-clipped kidney to increased AngII was ameliorated by nitric oxide (NO) release, suggesting that this is a protective mechanism (Helle et al., 2008). Additional early hypertension-induced changes in the renal tubules were identified by micro-dissection of visibly undamaged tubulointerstitial tissue from the non-clipped kidney. Proteomic analysis using mass spectrometry revealed the differential expression of over 300 proteins compared to control samples, with profibrotic Rho-signaling proteins being the most highly overrepresented (Finne et al., 2016). Such studies should help to identify additional biomarkers of early tubule damage, which in time could be used diagnostically. It should be noted, however, that the clipped kidney is not physiologically equivalent to an untreated (sham) control kidney; thus, the latter should always be included as a control when comparing clipped and non-clipped kidneys (Palm et al., 2008, 2010).

Despite complexities of the SHR, SHRSP and 2K1C hypertension models, a recent gene-expression profiling study revealed a common progression in hypertensive renal damage (Skogstrand et al., 2015). Of the 88 genes similarly regulated in all three models, 40 were also identified in gene-expression profiles from human fibrotic kidneys. This suggests that pathogenic pathways underlying kidney damage are conserved between rats and humans.

### Hypertensive models generated by genetic modification

Gene-knockout technology has only recently become available for the rat with the isolation of rat embryonic stem (ES) cells (see Glossary, Box 1) (Buehr et al., 2008; Li et al., 2008), which can be used as a tool for gene modification. The genetic tractability of the rat has also been greatly facilitated by genome-engineering technologies, such as zinc-finger nucleases (ZFNs) (Geurts et al., 2009), transcription activator-like effector nucleases (TALENs) (Tesson et al., 2011) and the CRISPR-Cas9 system (see Glossary, Box 1) (Li et al., 2013). Genome endonuclease technologies generate a sequence-specific DNA double-strand break, which is repaired by error-prone, non-homologous end-joining. Any insertions or deletions introduced at the target site cause missense or nonsense mutations. The PhysGen knockout program (http://pga.mcw.edu/) has utilized these technologies to generate a wide variety of knockout rat models in genes associated with cardiovascular or renal disease. One of the earliest ZFN-knockout rat models generated with a clear renal phenotype was the hypotensive renin-knockout rat (Moreno et al., 2011). Disruption of the renin gene caused profound disruption to normal kidney development. The inner renal medulla was morphologically rudimentary and there were signs of cortical interstitial fibrosis. These changes could be related to the concomitant reduction in AngII production, and support the assertion that the RAAS is essential for normal kidney development in mammals (Guron and Friberg, 2000).

Another rat knockout model that exhibits reduced renin levels is the Hsd2KO rat (Mullins et al., 2015). The enzyme 11-β-hydroxysteroid dehydrogenase type 2 (Hsd11b2) protects the MR from inappropriate activation by cortisol (corticosterone), in the kidney principal cell, by inactivating it to cortisone (11-dehydrocorticosterone). In this model, ZFN-induced knockout of the *Hsd11b2* gene causes inappropriate activation of the MR, leading to salt-sensitive hypertension, suppression of renin secretion, and hypokalemia (see Glossary, Box 1). This phenotype closely models the human syndrome of apparent mineralocorticoid excess (SAME). The rats exhibit severe renal injury, including protein casts and atrophic tubules, segmental glomerulosclerosis, tubule-interstitial fibrosis and proteinuria (Mullins et al., 2015). These are all features associated with chronic exposure to hypertension and with MR activation seen in human kidney disease (Ueda and Nagase, 2014). Interestingly, the Hsd2KO rat model demonstrates metabolic protection, including increased insulin sensitivity and reduced mesenteric fat accumulation, due to the depletion of the substrate for Hsd11b1 in adipose tissue. This suggests that treatment with MR inhibitors might reverse the adverse cardiovascular effects of SAME (which include hypokalemia, hypertension, proteinuria and end-organ damage), while promoting the beneficial metabolic effects of *Hsd11b2* inactivation (Mullins et al., 2015).

Salt-sensitive hypertension involves a complex feedback loop of salt appetite and sodium retention. Hsd11b2 in the murine brain triggers a central drive to consume salt (Evans et al., 2016). The rat Hsd2KO model offers a more robust platform to investigate the physiological mechanisms of central versus renal-centric salt sensitivity than is feasible in the mouse. Decreasing dietary salt consumption might reduce the burden of CKD in humans (McMahon et al., 2013). Intriguingly, an alternative, albeit more invasive, strategy to ameliorate salt-sensitive hypertension has been recently demonstrated. Renal medullary dysfunction in salt-sensitive Dahl rats (Dahl et al., 1962) was found to reflect a reduction in adult (CD133+) mesenchymal stem cells (MSCs) in the medulla. Injection of MSCs, but not of renal medullary interstitial cells, into the renal medulla attenuated immune-cell infiltration and sodium retention, and reduced systemic blood pressure (Hu et al., 2014). The rationale for using MSCs stems from numerous animal studies, which have demonstrated that these cells have protective effects in acute and chronic kidney injury models (Fleig and Humphreys, 2014; Wang et al., 2013).

The co-injection of single-strand oligonucleotides with ZFNs, TALENs or CRISPR-Cas9 components can be used to introduce targeted SNPs or to repair mutations, through homology-driven repair (HDR). Rapid improvements in CRISPR-Cas9 technology, using donor plasmids as HDR templates, have included the introduction of fluorescent reporters (Ma et al., 2014a), the one-step generation of a floxed allele (*loxP* sites flanking an exon) (Ma et al., 2014b) and conditional knockout using Cre-recombinase rat strains (see Glossary, Box 1) (Ma et al., 2014a). Recently, Wistar-Kyoto rats and SHRs that ubiquitously express GFP have been produced, using the Sleeping Beauty transposon system. These strains will prove useful for investigating cell fate and transplantation in the hypertensive kidney (Garcia Diaz et al., 2016).

The identification of genes such as *Ace*, *P2rx7* and *Hsd11b2*, or specific genetic variants or splice variants of genes, that seem to play key roles in moderating hypertensive damage, renal pathology and salt-sensitivity has the potential to enable future identification of individuals at risk of hypertensive kidney damage based on their genetic profile. With the availability of humanized transgenic models, Cre-*loxP* technology, reporter strains, gene knockouts and knock-ins, and the ability to correct candidate genes in mutant rat strains, many of the tools available to the mouse community are now available in the rat. Although the inherent problem of off-target events remain for genome-engineering technologies, targeting in rat ES cells and screening for clones free of off-target events remains a possibility. Thus, many more-refined and increasingly sophisticated rat models, which more closely recapitulate human renal pathology caused by hypertensive damage, can be expected in the future, and might help to predict targeted therapeutic response more faithfully.

## Models of diabetic nephropathy

Diabetic nephropathy (DN; see Glossary, Box 1) is the single most common cause of end-stage kidney disease in the western world (Saran et al., 2015). The use of reliable animal models of DN could greatly facilitate research by providing mechanistic insights into this disease to help identify novel therapeutic targets. These in turn could provide a platform for preclinical testing of such novel therapies. Unfortunately, one of the roadblocks to DN research is the lack of preclinical models that recapitulate important functional, structural and molecular pathological features of progressive human diabetic kidney disease. Although several rodent models of type 1 diabetes [streptozotocin (STZ)-induced (Cooper et al., 1988)] and type 2 diabetes [Zucker, Goto Kakizaki (Janssen et al., 2003)] have been employed to study DN (see Glossary, Box 1), these models fail to recapitulate all of the hallmarks of this disease as defined by the Diabetic Complications Consortium (DiaComp; https://www.diacomp.org/shared/validationcriteria.aspx). The inability of animal models to fully replicate human DN might explain why many therapies that have been beneficial in preclinical models of this disease have proven to be ineffective in clinical trials. For example, direct renin inhibitors were beneficial in reducing proteinuria in rodent models (Kelly et al., 2007). However, the absence of progressive renal failure in these models meant that the efficacy of these inhibitors in reducing renal function could not be tested. Human studies confirmed a beneficial effect of direct renin inhibitors on reducing proteinuria (Parving et al., 2008) but, importantly, they did not slow the rate of renal-function decline (Parving et al., 2012). Furthermore, the increased risk of hyperkalemia (see Glossary, Box 1) resulting from treatment with direct renin inhibitors in patients with impaired renal function (Parving et al., 2012) was not highlighted in the rodent models, where blood potassium levels remained normal.

Although hyperglycemia (see Glossary, Box 1) is a pre-requisite for the development of DN, hemodynamic factors play a substantial role in the progression of this disease. Individuals with advanced DN invariably have hypertension, and tight control of blood pressure is as important as glycemic control in slowing disease progression (Mogensen, 1998). Hypertension might not only be a consequence of nephropathy but a key driver of kidney disease in diabetes. Indeed, subtle abnormalities in blood pressure, such as loss of nocturnal dipping (see Glossary, Box 1), precede the onset of albuminuria (see Glossary, Box 1) in adolescents with type 1 diabetes (Lurbe et al., 2002). Furthermore, there are two case reports regarding individuals with longstanding diabetes, hypertension and unilateral renal artery stenosis (Berkman and Rifkin, 1973; Béroniade et al., 1987) whose conditions mimic the 2K1C rat model of hypertension. Autopsy findings in both cases revealed no pathological evidence of nephropathy in the kidney downstream of the arterial stenosis, despite severe nephropathy in the contralateral kidney. The implications of these findings are that unilateral renal artery stenosis might prevent the transmission of systemic hypertension to the kidney parenchyma and the subsequent development of nephropathy, even though both kidneys have been exposed to an equivalent degree of hyperglycemia and to increased AngII exposure. Thus, hyperglycemia or elevated angiotensin levels alone are insufficient to promote advanced DN; the development of hypertension is a prerequisite for disease progression. How hypertension interacts with hyperglycemia to promote nephropathy is unclear, but the application of cyclical stretch to mesangial cells cultured in high-glucose media increases the expression of pro-fibrotic genes, suggesting a role for increased mechanical strain (Gruden et al., 2000). In rat mesangial cells grown in high-glucose media, ATP and a P2X7 agonist dose-dependently increased ECM deposition and levels of transforming growth factor beta (TGFβ; a pro-fibrotic cytokine), whereas P2X7 inhibition attenuated the response (Solini et al., 2005), indicating the involvement of purinergic receptors.

Several approaches have been taken to recapitulate these important hemodynamic factors in rodent models of DN. In the 1980s, the Brenner group determined that a high-protein diet increased intra-glomerular pressure and promoted glomerular injury in diabetic rats and that these features could be successfully prevented by Ace inhibition (Zatz et al., 1986, 1985). These seminal studies led directly to clinical trials of ACE inhibitors in patients with DN, and they represent one of the best examples of how rodent models can be utilized to provide important mechanistic insights that subsequently lead to therapeutic advances. Indeed, ACE inhibitors have since become the mainstay of preventing the progression of renal disease in individuals with DN (Lewis et al., 1993). Conversely, many therapies that have been effective in animal models of DN that targeted hyperglycemia alone have proven unsuccessful in clinical trials (B.R.C., personal observation).

### Rat models of DN

Genetic models of hypertension have also been utilized to model progressive DN. The induction of diabetes with STZ leads to higher levels of albuminuria in SHRs than in rat strains with diabetes or hypertension alone (Cooper et al., 1988). Treatment with Ace inhibitors abrogates the increase in albuminuria in SHR strains. Activation of the RAAS plays a pre-eminent role in clinical DN. Therefore, a logical approach was to induce diabetes in *mRen2* rats (Kelly et al., 1998). The renin-dependent hypertension in *mRen2* rats accelerates the development of nephropathy, and this model has been used to study not only the role of the RAAS in DN, but also that of other pathways, including oxidative stress (Advani et al., 2009). It has been shown that sustained hyperglycemia causes increased tubular oxygen consumption due to mitochondrial dysfunction and reduced electrolyte transport efficiency (reviewed in Hansell et al., 2013). The onset of malignant hypertension in the *mRen2* model results in accelerated renal injury and in early mortality, which is atypical of the slowly progressive course observed in human diabetic kidney disease (Hartner et al., 2007). This problem was overcome by using *Cyp1a1mRen2* rats, where adjustment of I-3-C concentration in the diet controls the timing and severity of hypertension. Following induction of diabetes using STZ, the addition of 0.125% I-3-C resulted in a gradual increase in blood pressure, mimicking the evolution of hypertension in human DN (Conway et al., 2012). The hyperglycemia and hypertension synergized to promote a 500-fold increase in albuminuria, and caused moderate glomerulosclerosis and tubulointerstitial fibrosis – all features of moderately advanced human DN. However, there was no significant decline in renal function in this model, and some key pathological features of DN, such as arteriolar hyalinosis (see Glossary, Box 1), were not observed.

Microarray and RNA-sequencing technologies provide a non-biased view of gene expression changes. Thus, comparing transcriptomic changes in DN patients with rat models of the disease might reveal common disease mechanisms, identify relevant biomarkers and therapeutic targets, and enable the rational selection of the rodent model that most closely recapitulates changes seen in DN kidneys. Up to 50% of genes that were differentially expressed in the tubulointerstitial compartment of the kidney in human DN (Lindenmeyer et al., 2007) were also similarly up- or downregulated in the renal cortex of hyperglycemic and hypertensive *Cyp1a1mRen2* rats (Conway et al., 2012). For example, one downregulated gene in both the rat model and in the kidneys of individuals with DN was epidermal growth factor (EGF). Urinary EGF levels reflect renal EGF expression, and subsequent studies confirmed that low levels of urinary EGF excretion predict a poor renal outcome in individuals with DN and with other CKDs (Betz et al., 2016; Ju et al., 2015). Hence, non-biased transcriptomic approaches could be used to identify as-yet-unknown prognostic biomarkers for therapeutic targets or to recruit high-risk individuals for clinical trials. Such transcriptomic datasets should be made freely available on databases such as Geodataset (http://www.ncbi.nlm.nih.gov/gds/) or Nephroseq (https://www.nephroseq.org), as this will enable researchers to select the model in which their pathway of interest is differentially activated in a similar manner to human disease. Such ‘precision modeling’ could improve the chances of translating findings made in rodent models to the clinic.

Although the natural history of DN is one of inexorable progression towards end-stage kidney disease, the tight control of blood glucose and blood pressure can lead to the regression of albuminuria in up to 50% of individuals with DN (Perkins et al., 2003). More remarkably, regression of established glomerulosclerosis and tubulointerstitial fibrosis has been observed in individuals with moderately advanced DN who achieve sustained normoglycemia after receiving a pancreas transplant (Fioretto et al., 1998, 2006), although this takes up to 10 years to become evident. The pathways that promote regression remain poorly understood, largely because serial biopsies are rarely performed in individuals who are responding to treatment.

Rodent models provide insights into mechanisms of injury, regeneration and repair. The *Cyp1a1mRen2* rat model of DN is particularly useful in this regard because hypertension can be induced and then blood pressure normalized by adding and then removing dietary I-3-C; inserting subcutaneous insulin implants can also control STZ-induced hyperglycemia. In one study, 28 weeks of hyperglycemia and hypertension (the injury phase) were followed by tight glycemic and blood pressure control for an additional 8 weeks (the reversal phase), resulting in the partial regression of albuminuria (Conway et al., 2014). Microarray analysis of the renal transcriptome during both the injury and reversal phases revealed ∼650 genes that were upregulated during injury, almost 100 of which reverted to control levels following reversal of hyperglycemia and hypertension. This gene set was enriched for genes that encoded ECM proteins, fibroblast markers and acute-phase reactants, indicating that the tight control of glucose and blood pressure might suffice to switch off the formation of new scar tissue. This was supported by the finding that there was no further increase in the severity of glomerulosclerosis or tubulointerstitial fibrosis during the 8-week reversal phase. In addition, many genes of unknown function, which reverted to control levels during repair, might be implicated in the fibrotic- or acute-phase response and hence they merit further investigation. Conversely, almost 400 genes remained significantly upregulated despite the normalization of blood glucose and blood pressure. This gene set was enriched for genes that encoded proteins implicated in innate and adaptive immunity, in particular pro-resolution macrophages and regulatory T cells, suggesting that attempts at repair have been initiated. Although glomerulosclerosis and tubulointerstitial fibrosis did not reduce during the reversal phase, this was to be expected given the protracted period required for regression of fibrosis following pancreas transplantation in humans (Fioretto et al., 2006). Permanent or long-term upregulation of some of these genes might be responsible for the salt sensitivity observed in I-3-C-induced rats (Howard et al., 2005).

Bilateral renal denervation has emerged as a potential treatment for multiple-drug-resistant hypertension in individuals with bilateral single renal arteries, but results from recent clinical trials have questioned its efficacy for individuals with secondary (or accessory) renal arteries (Bhatt et al., 2014; Hering et al., 2016; Khan et al., 2014). When bilateral renal denervation was performed in the *mRen2*/STZ rat model, it reduced signs of renal pathology, albuminuria and the expression of fibrotic markers. This suggests that renal denervation might attenuate renal injury in DN (Yao et al., 2014), presumably with similar caveats regarding efficacy.

In summary, rat studies can mimic many of the features of human DN, including progressive proteinuria, key pathological features such as glomerulosclerosis and tubulointerstitial fibrosis, and the activation of many pathways that are implicated in human DN. However, none fully recapitulate human DN, with few exhibiting arteriolar hyalinosis and a progressive decline in renal function. Rat models have highlighted the benefits of Ace inhibitors and the prognostic value of EGF in the treatment of DN. A comparison of the results from microarray and RNA-sequencing technologies in rodent models and human DN will continue to identify new candidates for therapeutic interventions to prevent kidney damage or to enhance repair and regeneration.

## Models of acute and chronic kidney disease

AKI affects multiple cell types in the kidney, including endothelial and tubular cells, which are adversely affected by hypoxia. It is not clear whether hypoxia (the reduction of tissue oxygen supply to below physiological levels) or re-oxygenation (increased exposure to oxygen, as seen with reperfusion following ischemia) causes AKI, but it is associated with altered intra-renal microcirculation and oxygenation (Rosenberger et al., 2006). Ischemia-reperfusion injury (IRI; see Glossary, Box 1) is extensively used as a model of AKI, but hypoxic damage predominantly affects proximal tubule segments in the outer stripe of the outer medulla and might not recapitulate human AKI, which often includes medullary oxygen insufficiency. Damage to the thick ascending limb is attenuated following IRI, probably because the reduced solute transport leads to improved oxygenation of the distal tubule (Rosenberger et al., 2006). Following acute IRI, the vascular function of rats remains impaired for several days (Conger et al., 1991). The pericyte (see Glossary, Box 1) detaches from the endothelium under pathological conditions, leading to microvascular rarefaction and hypoxia (Schrimpf et al., 2014). Pericytes might contribute to the pool of scar-forming myofibroblasts (see Glossary, Box 1) (Kramann and Humphreys, 2014), making them key to both regeneration and the development of fibrosis (Schrimpf and Duffield, 2011), although myofibroblasts can also arise from other sources (Falke et al., 2015; Micallef et al., 2012).

Agents affecting both cortical and medullary blood flow and oxygen tension include radio-contrast agents (Heyman et al., 1991), endotoxin [sepsis (Heyman et al., 2000)] and NO inhibitors (Brezis et al., 1991). Together with non-steroidal anti-inflammatory drugs, which cause a selective reduction in medullary blood flow and tissue oxygenation, these could provide better models of AKI and could enable investigation of hypoxia-inducible factors, adaptive responses and potential therapies (Rosenberger et al., 2006). The development of rat models should enhance our understanding of AKI and help to design therapeutic strategies to block maladaptive responses.

Pre-existing CKD affects the severity of AKI in humans and their recovery (Liangos et al., 2006). This has been experimentally modeled in rats using the renal-mass-reduction model of CKD with an additional induced IRI. CKD develops in the 5/6th nephrectomy rat model (in which the 5/6th of renal mass is surgically ablated; see Table 1). When AKI is induced in this model via IRI, a disproportionate number of regenerating tubules fail to re-differentiate. This is associated with significant loss of tubular VEGF expression and with substantial capillary rarefaction. Defective tubules also have pro-fibrotic properties that increase tubulointerstitial fibrosis (Polichnowski et al., 2014). Further investigation of this model will provide a greater understanding at the molecular level of the AKI to CKD transition seen in humans.

Reporter rats should prove invaluable for mechanistic studies and for the identification of the molecular pathways and cell lineages involved in kidney disease (Garcia Diaz et al., 2016). The creation of reporter transgenic rats has allowed the mapping of cells that contribute to renal fibrosis and the testing of novel anti-fibrotic agents on key pro-fibrotic pathways (Terashima et al., 2010). Using transgenic rats carrying a luciferase reporter gene under the control of rat α_1_(I) collagen and rat α_2_(II) collagen, the anti-fibrotic effects of inhibiting TGFβ signaling (using a TGFβR1 inhibitor) and AngII signaling [using an AngII-receptor blocker (ARB), olmesartan] were examined (Terashima et al., 2010). This study revealed that ARBs had an anti-fibrotic effect, independent of hemodynamic effects, in the unilateral ureteral obstruction (UUO) model of rapid renal fibrosis (see Table 1), which induces a marked change in renal perfusion.

Rat models of AKI and CKD have been used as a platform to test potential new therapies, including novel anti-fibrotic agents. FT011 is a derivative of the anti-allergy drug Tranilast (Miyazawa et al., 1995), and it inhibits the proliferative actions of TGFβ and platelet-derived growth factor (PDGF). FT011 stemmed the decline in GFR in the 5/6th nephrectomy model of progressive CKD (see Table 1) and reduced proteinuria and structural injury (Gilbert et al., 2012). In the diabetic, hypertensive *mRen2*/STZ model, FT011 markedly attenuated the development of proteinuria, as well as reducing fibrosis in both the glomerulus and tubulointerstitium, and interstitial macrophage infiltration, but GFR was unaffected (Gilbert et al., 2012).

In a rat model of aristolochic-acid-induced nephropathy, the neutralization of TGFβ with anti-TGFβ antibody improved renal function and reduced acute tubular necrosis, interstitial inflammation, vascular rarefaction and myofibroblast accumulation (Pozdzik et al., 2016). The disruption of proximal tubule organelle ultrastructure was also prevented. However, these findings have not translated to the clinic; agents that block TGFβ and retard CKD have failed to improve renal function despite the promising preclinical results (Lee et al., 2015). These findings again support the observation that animal models typically recapitulate only part of the human condition – particularly CKD and its progression to ESRD. Animal models such as the UUO rat, used as a model of renal fibrosis, can be studied for a few weeks at most, whereas, in humans, these conditions usually develop over many years. Pathways that are important initially might not be as important in the pathophysiology of later disease and could explain the lack of translation of successful preclinical compounds.

Studies performed in various transgenic rat models have led to new insights into glomerulosclerosis, and in particular into the role of the podocyte (see Glossary, Box 1). A direct causative relationship exists between the degree of podocyte depletion and the development of proteinuria and glomerulosclerosis (Kim et al., 2001; Wharram et al., 2005). However, the mechanisms by which podocyte depletion can lead CKD to progress to end-stage kidney disease are poorly understood.

To examine the effect of podocyte depletion, the human diphtheria toxin receptor (hDTR) was specifically expressed in podocytes, generating the hDTR Fischer rat model (see Table 1), which has histopathological features commonly seen in the human disease focal segmental glomerulosclerosis (FSGS; see Glossary, Box 1), including mesangial expansion, segmental and global sclerosis (Wharram et al., 2005). These features occur in proportion to the degree of podocyte depletion. Although a return to normal glomerular architecture over time did not occur, once the glomerulus was destabilized by a critical degree of podocyte loss, the continuous infusion of an ACE inhibitor (enalapril) and ARB (losartan) was found sufficient to stabilize the glomeruli. The reno-protective effect of ARBs is not through blood pressure reduction alone and seems to be due to a direct effect on the podocyte (Fukuda et al., 2012b; Wharram et al., 2005).

Another transgenic Fischer rat model, this time expressing a dominant-negative phosphorylation site mutant of *AA-4E-BP1*, the eukaryotic translation initiation factor binding protein 1 (*EIF4EBP1*) transgene (see Table 1), has been used to examine the effect of growth on podocyte failure (Fukuda et al., 2012a). Driven by the podocin promoter, the *EIF4EBP1* transgene encodes a member of the mammalian target of rapamycin complex 1 (mTORC1) pathway, which is a key determinant of the cellular hypertrophic response, driven by the podocin promoter. Transgenic *AA-4E-BP1* rats have normal kidney histology with no proteinuria below 100 g body weight, but develop end-stage renal disease by 12 months. The observed proteinuria and glomerulosclerosis were linearly related to body weight increases and transgene dose. Histological observations revealed bare areas of glomerular basement membrane, where podocyte foot processes had pulled apart, and consequent adhesion to the Bowman capsule. In the *AA-4E-BP1* model, it seems that proteinuria develops through mechanical failure of the podocyte epithelial layer. This mechanism of podocyte depletion is different from direct podocyte damage and death. It also provides a mechanistic explanation for a separate group of diseases that lead to global glomerulosclerosis or focal segmental glomerulosclerosis (see Glossary, Box 1) in childhood and obesity (Fukuda et al., 2012a), suggesting that limiting calorie intake could be beneficial in reducing the severity of the human condition. With additional developments, such as intravital imaging (Peti-Peterdi et al., 2016) and visualization of calcium dynamics (Szebenyi et al., 2015) to observe podocyte function/glomerular injury processes in real time, a deeper understanding of the mechanisms that lead to the development of renal pathology should identify novel therapeutic targets.

Novel monogenic rat models of glomerulosclerosis have also been generated, such as the TGR(hET-2)37 rat model, which expresses high levels of human endothelin-2 (ET2) in the kidney (Hocher et al., 1996). These rats develop blood-pressure-independent glomerulosclerosis, which demonstrates that the human *ET2* gene can have a blood-pressure-independent, growth-promoting effect on the rat glomerulus.

Apoptosis is a key feature of the progression of CKD. Recently, ouabain, which is a cardiotonic steroid, has been found to have anti-apoptotic actions. Chronic ouabain treatment of rats with passive Heymann nephritis [PHN; a model of human membranous nephropathy, a slow progressive proteinuric kidney disease (Salant et al., 1979)] prevented the loss of podocytes, reduced the level of apoptotic proximal tubule cells and reduced renal fibrosis (Burlaka et al., 2016). Ouabain might represent a novel therapy that could potentially protect against apoptosis and prevent the loss of functional tissue in chronic proteinuric kidney disease.

The anti-Thy1.1 model of glomerulonephritis is an experimental rat model that mimics human antigen-triggered, immune-induced mesangio-proliferative glomerulonephritis (MPGN; see Glossary, Box 1), such as IgA nephropathy. This well-characterized model of glomerular injury has been used to investigate molecular mechanisms of mesangial proliferation. Proteomic studies have revealed several proteins that show altered expression in this model (Nazeer et al., 2009), particularly the four and a half LIM domain protein 2 (FHL2), which increases mesangial cell proliferation *in vitro* (Lu et al., 2012) and could represent a new target for treating MPGN. This model has proven to be useful in identifying key stress-induced microRNAs, such as miR-21 and miR-214 (Denby et al., 2011), which are upregulated during renal injury. These microRNAs have since been found to be differentially expressed in human biopsies of individuals with IgA nephropathy, and their upregulation correlates linearly with renal fibrosis (Hennino et al., 2016), demonstrating the translational relevance of this model.

Other rat models of glomerulonephritis include the nephrotoxic nephritis (NTN) model (see Table 1), which established that levels of the P2X7 receptor protein are increased in the glomerulus. This correlates with increased glomerular P2X7 in human biopsy samples from patients with nephritis due to lupus (Turner et al., 2007). In the rat NTN model, the P2X7 antagonist A-438079 prevented antibody-mediated glomerulonephritis through reduced inflammatory damage due to a reduction in macrophage infiltration into the glomerulus (Taylor et al., 2009).

Rat models have proved to be invaluable in the field of regenerative cell therapy for renal disease. The potential of bone-marrow-derived MSCs to accelerate healing has been demonstrated in several rat models of hypertension (as discussed above) and of renal disease, including in the anti-Thy1.1 model (Li et al., 2006), the 5/6th nephrectomy model of progressive CKD (Cavaglieri et al., 2009; Choi et al., 2009) and in an AKI model induced by cisplatin (Urt-Filho et al., 2016). MSCs might reverse AKI by a paracrine mechanism rather than by MSC transdifferentiation. Intravenous injection of microvesicles, released from cultured human MSCs, inhibited tubular apoptosis and stimulated regeneration (Gatti et al., 2011). The renoprotective effect was lost if microvesicles were pre-treated with RNAse, or if the pro-angiogenic microRNAs, miR-126 and miR-296, were depleted. This suggests that the miRNAs, delivered by microvesicles, are able to reprogram hypoxic resident renal cells (Cantaluppi et al., 2012). Importantly, MSCs taken from either the 5/6th nephrectomy model or the adenine-induced nephropathy model and transplanted into the anti-Thy1.1 model failed to induce healing. Both CKD and uremia adversely affected transplanted MSCs, which exhibited cellular senescence (Klinkhammer et al., 2014). This result brings into question the use of autologous MSCs for the treatment of CKD.

In summary, AKI and CKD share a spectrum of renal pathologies. The identification of early biomarkers could allow the practitioner to harness adaptive repair and regenerative mechanisms, and prevent the maladaptive profibrotic pathways. A better understanding of the roles of, and of the potential cross-talk between, pericytes, myofibroblasts, tubular epithelium and podocytes is key to developing new therapies, and the rat is well placed to deliver such advances.

## Renal transplantation

Renal transplantation was first performed in the rat over 50 years ago. Although the microsurgical techniques involved remain challenging, they are more readily mastered in rats than in mice. Several different combinations of inbred and outbred rat strains can be used to model various complications of renal transplantation, including IRI, acute rejection and chronic allograft nephropathy (CAN; see Glossary, Box 1) (Shrestha and Haylor, 2014). Renal transplantation from a Fischer donor to a Lewis recipient is the most common model of CAN in rats (White et al., 1969). Fisher and Lewis rat strains differ partially at the major histocompatibility loci (MHC) I and II, and this weak histocompatible combination results in CAN in the absence of immunosuppression (Hancock et al., 1992; Paul et al., 1998). Ace inhibition can limit kidney damage in this transplant model (Noris et al., 2003), which has also been used to assess the development of alloimmunity (de Heer et al., 1994), the efficacy of immunosuppressants (Chandraker et al., 1998), non-immune therapies (Magee et al., 1999) and the development of fibrosis in the graft (Jain et al., 2000). The small molecule BB3 is a hepatocyte growth-factor mimetic, and studies in an IRI-induced rat model of AKI revealed that BB3 protected the kidney from tubular apoptosis and necrosis (Narayan et al., 2016). These data form the basis of a clinical trial using BB3 in kidney-transplant recipients who present with delayed graft function.

Allograft and isograft renal transplantation can also be used to determine the relative importance of intrinsic renal cells versus bone-marrow-derived cells in the pathogenesis of a wide range of renal diseases. *Ex vivo* injection of MSCs into the kidney prior to transplantation proved beneficial, whereas systemic injection of MSCs failed to improve recipient survival (Iwai et al., 2014). Recent improvements in the ability to genetically manipulate rats open up an exciting new area of research for renal transplantation studies (Doorschodt et al., 2014).

## Conclusions and future perspectives

Disparities between animal models and human disease might have resulted in promising preclinical therapies failing to be effective in clinical trials. Recent developments in genome engineering and transcriptomic profiling now allow the researcher to design and refine models, to more closely interrogate specific aspects of renal disease. The rat has and will continue to play a major role in the identification of key genes that increase disease susceptibility, of early biomarkers that highlight disease progression, and of genes, pathways and cells that are fundamentally involved in kidney regeneration or damage.

As highlighted in this Review, hypoxia, AngII, ACE and P2X7 play key roles in many aspects of kidney damage, placing them at the forefront of therapeutic targets to be explored using rat models. Given the complex nature of, for example, human *P2X7* transcripts, humanization of the rat could help to identify which isoforms are disease-promoting, and could aid in the development of novel treatment strategies.

Of particular interest is the application of MSC technology to the treatment of AKI, CKD and renal transplantation. A number of MSC-based clinical trials have been set up, despite safety concerns raised by animal studies (Kunter et al., 2007). In a rat model of glomerulonephritis, MSCs produced a short-term improvement, but ultimately differentiated into intraglomerular adipocytes, resulting in glomerulosclerosis (Kunter et al., 2007). Enhanced recruitment of endogenous MSCs or the use of cell-free cocktails of secreted factors might be preferable approaches (Kunter et al., 2011).

It is important to note that the ‘treatment’ of kidney disease might not lead to repair of all aspects of organ damage. However, the complexity of renal pathologies means that better design and use of rat models as a resource could ultimately result in stratification of diagnosis and tailored therapy.
